# Incidence, characteristics and clinical relevance of acute stroke in old patients hospitalized with COVID-19

**DOI:** 10.1186/s12877-021-02006-2

**Published:** 2021-01-14

**Authors:** Aline Mendes, François R. Herrmann, Laurence Genton, Christine Serratrice, Emmanuel Carrera, Maria Isabel Vargas, Gabriel Gold, Christophe E. Graf, Dina Zekry, Max Scheffler

**Affiliations:** 1 Division of Geriatrics, Department of Rehabilitation and Geriatrics, University Hospitals of Geneva and Faculty of Medicine, Chemin du Pont-Bochet 3, 1226 Thônex, Geneva, Switzerland; 2 Unit of Clinical Nutrition, University Hospitals of Geneva and Faculty of Medicine, Geneva, Switzerland; 3 Division of Internal Medicine for the Aged, Department of Rehabilitation and Geriatrics, University Hospitals of Geneva and Faculty of Medicine, Geneva, Switzerland; 4 Division of Neurology, Department of Neurosciences, University Hospitals of Geneva and Faculty of Medicine, Geneva, Switzerland; 5 Division of Neuroradiology, Diagnostic Department, University Hospitals of Geneva and Faculty of Medicine, Geneva, Switzerland; 6 Division of Internal Medicine and Rehabilitation, Department of Rehabilitation and Geriatrics, University Hospitals of Geneva and Faculty of Medicine, Geneva, Switzerland; 7 Division of Radiology, Diagnostic Department, University Hospitals of Geneva, Geneva, Switzerland

**Keywords:** COVID-19, Stroke, Cerebrovascular, Ischemic stroke, Hemorrhagic stroke

## Abstract

**Background:**

Stroke in the course of coronavirus disease (COVID-19) has been shown to be associated with more severe respiratory symptoms and higher mortality, but little knowledge in this regard exists on older populations. We aimed to investigate the incidence, characteristics, and prognosis of acute stroke in geriatric patients hospitalized with COVID-19.

**Methods:**

A monocentric cross-sectional retrospective study of 265 older patients hospitalized with COVID-19 on acute geriatric wards. 11/265 presented a stroke episode during hospitalization. Mortality rates and two-group comparisons (stroke vs non-stroke patients) were calculated and significant variables added in logistic regression models to investigate stroke risk factors.

**Results:**

Combined ischemic and hemorrhagic stroke incidence was 4.15%. 72.7% of events occurred during acute care. Strokes presented with altered state of consciousness and/or delirium in 81.8%, followed by a focal neurological deficit in 45.5%. Ischemic stroke was more frequently unilateral (88.8%) and localized in the middle cerebral artery territory (55.5%). Smoking and a history of previous stroke increased by more than seven (OR 7.44; 95% CI 1.75–31.64; *p* = 0.007) and five times (OR 5.19; 95% CI 1.50–17.92; *p* = 0.009), respectively, the risk of stroke. Each additional point in body mass index (BMI) reduced the risk of stroke by 14% (OR 0.86; 95% CI 0.74–0.98; *p* = 0.03). In-hospital mortality (32.1% vs. 27.3%; *p* > 0.999) and institutionalization at discharge (36.4% vs. 21.1%; *p* = 0.258) were similar between patients with and without stroke.

**Conclusion:**

Incident stroke complicating COVID-19 in old patients was associated with active smoking, previous history of stroke, and low BMI. Acute stroke did not influence early mortality or institutionalization rate at discharge.

**Supplementary Information:**

The online version contains supplementary material available at 10.1186/s12877-021-02006-2.

## Introduction

Acute cerebrovascular complications have been reported through the course of bacterial and viral infections in both young and older patients [1, 2]. Multiple mechanisms may explain the phenomenon, such as a higher prothrombotic state induced by systemic inflammation, an increased incidence of arrhythmia, for example atrial fibrillation, and hypoxia leading to cerebral hypoperfusion, and possibly direct virus-induced vasculitis or endothelitis [3, 4].

Different neurological complications associated with severe acute respiratory syndrome coronavirus 2 (SARS-CoV-2) infection have been described, such as ischemic and hemorrhagic stroke, seizures, meningitis, encephalitis, and Guillain-Barre syndrome [5–8]. A recent systematic review polled 135 cases of ischemic stroke in patients with coronavirus disease 2019 (COVID-19), showing a stroke incidence varying from 0.9 to 2.7% [9]. Notably, acute stroke in the course of COVID-19 was associated with higher severity of respiratory symptoms and mortality in the acute phase.

However, the majority of studies available describe younger populations and little knowledge exists on acute stroke in old and very old patients with COVID-19. The overall incidence of stroke increases with age, as the general prevalence of cerebrovascular risk factors such as hypertension, diabetes, dyslipidemia, and atrial fibrillation. Likewise, older patients with COVID-19 present worse disease outcomes than younger patients, with increased mortality [10], having led to characterize them as a group at risk for more severe disease course and complications.

This study aimed to describe the incidence, and clinical and imaging characteristics of stroke, as well as their relationship with early mortality and destination at discharge, in a population of old and very old patients hospitalized with COVID-19**.** We hypothesized that the incidence of stroke in our COVID-19 population was higher than the one described in the literature, and that outcomes were worse.

## Methods

### Design, setting and population

This monocentric cross-sectional retrospective study analyzed patients hospitalized in geriatric wards of Geneva University Hospitals. Hospitalized patients with COVID-19 had one or more of these clinical features: a) pneumonia with a severity assessed by the CURB-65 score ≥ 2, b) new dependence on oxygen or increase of oxygen needs, c) a respiratory rate ≥ 20 breaths/minute, d) a decompensated chronic disease, e) severely altered general state of health, f) deteriorating clinical course.

We analyzed data from a total of 265 patients hospitalized with SARS-CoV-2 infection between March 13th, 2020 and May 17th, 2020. The geriatric hospital is part of Geneva University Hospitals, which were responsible during the first wave of the COVID-19 pandemic for hospitalizing all patients of its served population of approximately 500,000. This study was carried out in accordance with the STrengthening the Reporting of OBservational studies in Epidemiology (STROBE) statement. This study was authorized by the institutional board of the University Hospitals of Geneva and accepted by the Geneva’s committee for research ethics (project ID: 2020–00819).

### Data collection

Data regarding demographics, comorbidities, clinical symptoms, and laboratory analyses were retrospectively collected by one research nurse. In general, all information was obtained within the first 24 h of hospitalization in an acute ward. The geriatric hospital’s wards are managed by medical staff trained in internal medicine and geriatrics and can provide general medical care, such as intravenous pharmacotherapy and non-invasive oxygen support (by nasal cannula and face mask, allowing an increase of the fraction of inspired oxygen from 24 to 65%). Discharge criteria were an improvement of acute illness with subsequent transfer to a rehabilitation ward, or discharge to home or to a nursing home. For patients presenting worsening symptoms and unfavorable evolution, end-of-life care was implemented on the ward with consultation of palliative mobile teams. The diagnosis of COVID-19 was defined as a positive reverse transcription polymerase chain reaction (RT-PCR) test for SARS-CoV-2 on a nasopharyngeal swab. Patients with negative virus detection by RT-PCR (18/265; 6.8%) but a high clinical suspicion of disease were also diagnosed with COVID-19 [11].

The Functional Independence Measure (FIM) was performed by a nurse, based on observation during the first 24 h after hospital admission. It reflects functional status and physical function, with a point score ranging from 18 to 126, higher scores corresponding to better functionality [12]. The Clinical Frailty Score (CFS) was calculated by the physician in charge, reflecting the condition of older patients before hospitalization [13]. Higher CFS scores (from 5 to 8) correspond to more severe degrees of frailty, with a terminally ill patient assigned the highest score of 8. The Cumulative Illness Rating Scale-Geriatric (CIRS-G) was performed at admission, measuring a patient’s comorbidity burden by taking into account chronic diseases as well as the severity of acute illnesses, with higher scores representing a higher overall disease burden [14]. The severity of respiratory symptoms was assessed using the Pneumonia Severity Index (PSI) [15] and the abovementioned CURB-65 [16], which varies from 0 to 5, with higher scores being associated with higher mortality. For patients diagnosed with stroke, we computed the Modified Ranking Score (MRS) [17], which assesses functional recovery after a stroke event, as well as the CHA_2_DS_2_-VASc [18] and HAS-BLED [19] scores for thromboembolic and bleeding risks, respectively.

Cerebrovascular complications were defined as a diagnosis of stroke with neuroimaging confirmation by CT or MRI, including both ischemic stroke and intracranial hemorrhage. We included all cases of stroke having occurred during hospitalization, between the moment of COVID-19 diagnosis and hospital discharge or death. Hence, stroke diagnoses documented during acute and rehabilitation hospital stays were included in the analysis. For all stroke cases identified, an extensive review of the electronic medical record and imaging results was performed by a geriatrician and, for the purpose of this study, all images were reviewed by the same neuroradiologist.. Stroke cases were classified with respect to their localization, extent of ischemia or hemorrhage, and vascular territory [20]. In general, all patients that were suspected to present a stroke underwent standardized neuroimaging by CT or MRI, routinely performed according to in-house protocols.

### Statistical analysis

Categorical variables are expressed as numbers and proportions, while continuous variables appear as means and standard deviations. A two-group comparison (patients with and without stroke) was performed using Fisher’s exact test and independent t-test for categorical and continuous variables, respectively. For the CHA_2_DS_2_-VASc and HAS-BLED scores from the stroke group, results are expressed as medians with minimum and maximum values. Because of the low number of stroke occurrences, only univariate logistic regression models were calculated to identify stroke predictors. The variables selection was based on statistically significant differences observed in the two-group comparison (patients with and without stroke). Furthermore, for the significant variables, we performed a trend analysis to study the relationship with stroke occurrence [21].

Statistical analysis was performed using Stata software version 16.1 (Stata Corp., College Station, TX, USA).

## Results

### Characteristics of the study population and stroke risk factors

**Fig. 1:**
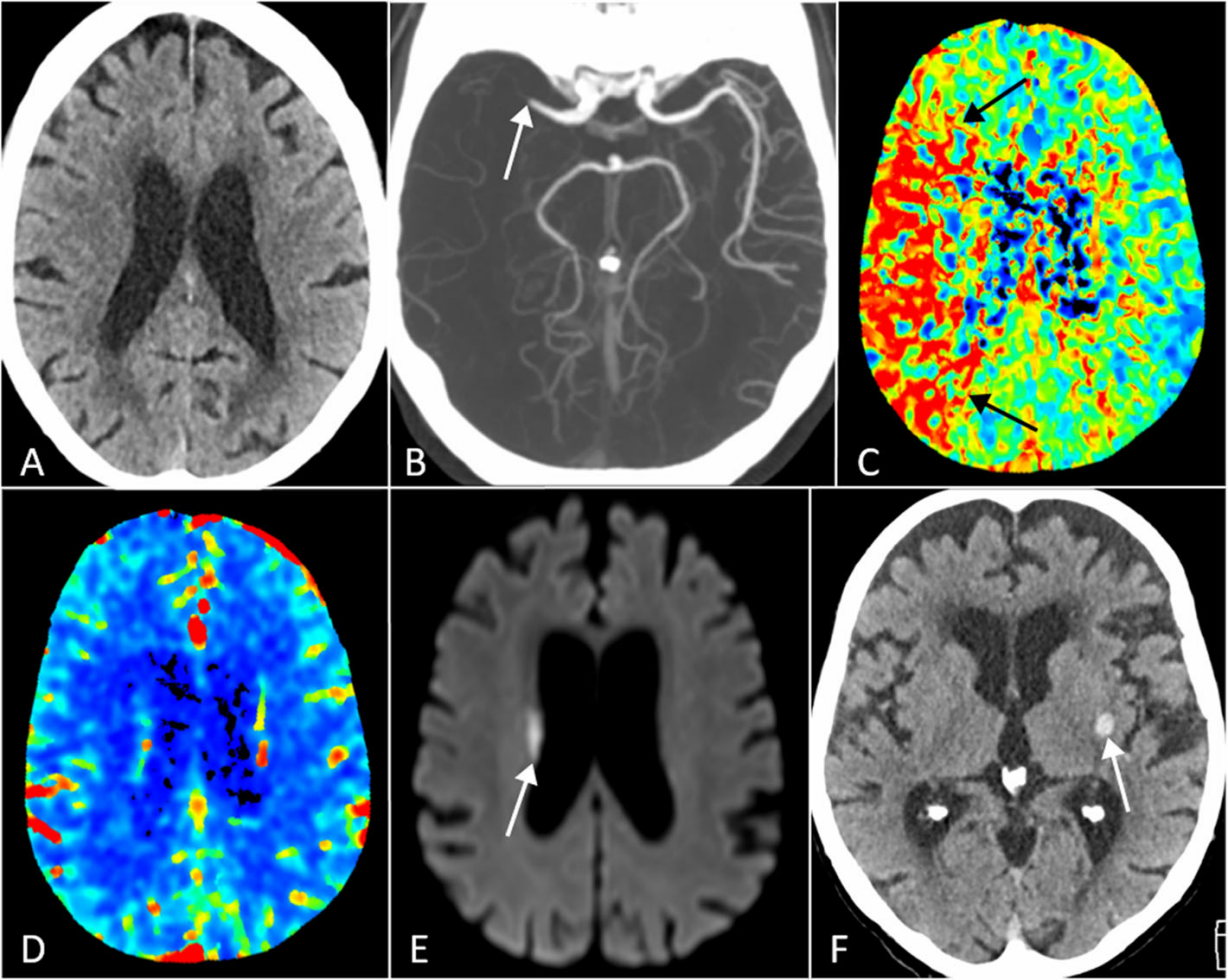
Illustrative images of neurologic complications in older patients hospitalized with COVID-19 pneumonia. **a**, **b**, **c**, **d**, and **e**, CT and MRI images of an 89-year-old woman with left-sided hemiplegia. **a**, Non-contrast head CT did not show early CT signs of ischemia. **b**, CT angiogram showed occlusion of the right middle cerebral artery (arrow). **c**, Perfusion CT mean transit time (MTT) images showed prolonged MTT in the entire right middle cerebral artery territory (arrows), whereas perfusion CT derived cerebral blood volume (**d**) remained symmetric between both hemispheres. **e**, Diffusion-weighted image of MRI study obtained one day later and after intravenous thrombolysis showed a small area of infarction in the right corona radiata (arrow). **f**, Non-contrast head CT image of an 84-year-old woman with obnubilation showed an isolated focus of intraparenchymal hemorrhage in the left lentiform nucleus (arrow). No evidence of underlying vascular or tumoral pathology was found on subsequent MRI (not shown)

The study population consisted of 265 patients with a mean age of 85.9 ± 6.5 years, 43% were male, and the in-hospital mortality rate was 32.1%. Acute stroke was confirmed in 11 (4.15%) patients (Fig. 1). Mortality was similar between patients with (32.1%) and without stroke (27.3%, *p* > 0.999), and there were no differences regarding age, sex, or length of stay in an acute ward.

**Fig. 2:**
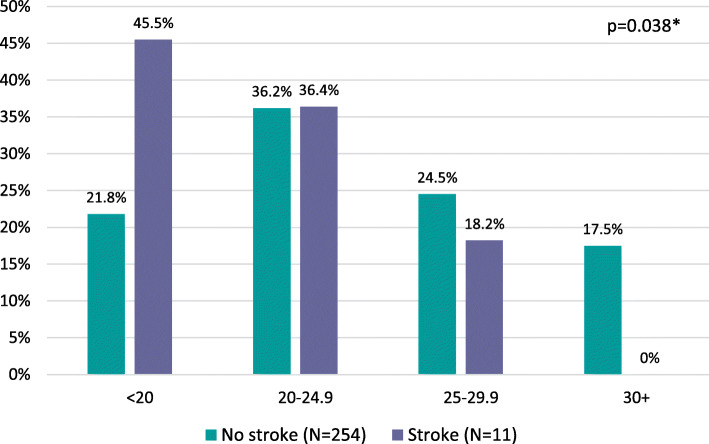
Distribution of body mass index (kg × m^−2^) categories in patients with and without stroke. *p-value corresponds to trend analysis of stroke incidence across body mass index categories [21]

Stroke patients had a higher prevalence of active smoking (27.3% vs. 4.8%; *p* = 0.019), as well as a history of previous stroke (45.5% vs. 13.8%; *p* = 0.014). On the other hand, tiredness was less frequently reported in the group of stroke patients (9.1% vs. 50.2%; *p* = 0.010). Interestingly, stroke patients had a lower BMI than those without stroke (20.7 ± 3.5 vs. 24.9 ± 6.4; *p* = 0.002). While their mean BMI value was within the “normal” range, 45.5% of calculated BMIs were in the underweight range (< 20 kg × m^− 2^), while no patient with acute stroke was classified as obese (BMI ≥30 kg × m^− 2^) (Fig. 2). The trend towards a lower BMI in the stroke group was statistically significant (*p* = 0.038).

There were no differences regarding other cerebrovascular risk factors, except for dyslipidemia, which was more frequent in stroke patients, but with a borderline statistically significant *p*-value (63.6% vs. 33.2%; *p* = 0.051). Similarly, we did not observe differences in comorbidity burden, functional status, frailty, the severity of COVID-19, or the occurrence of other thromboembolic events between the two groups (Table 1).

**Table 1:** Characteristics of patients with ischemic and hemorrhagic types of acute stroke compared to those without stroke

Characteristics	Total	No stroke	Stroke	***p*** value
	*N* = 265	*N* = 254	*N* = 11	
Age, years	85.9 ± 6.5	85.8 ± 6.6	87.1 ± 4.5	0.398
Length of stay in acute care, days	12.6 ± 7.7	12.7 ± 7.7	11.9 ± 7.8	0.645
Male sex	114 (43.0%)	109 (42.9%)	5 (45.5%)	> 0.999
Help in ADL/IADL	127 (47.9%)	121 (47.6%)	6 (54.5%)	0.762
Mortality	85 (32.1%)	82 (32.3%)	3 (27.3%)	> 0.999
Number of medications	7.4 ± 4.1	7.4 ± 4.1	6.4 ± 3.0	0.286
Heart failure	93 (36.5%)	90 (36.9%)	3 (27.3%)	0.750
COPD	30 (11.4%)	27 (10.7%)	3 (27.3%)	0.117
Active smoking	15 (5.7%)	12 (4.8%)	3 (27.3%)	0.019
Kidney disease	74 (28.2%)	70 (27.9%)	4 (36.4%)	0.510
Diabetes under treatment	63 (24.0%)	60 (23.9%)	3 (27.3%)	0.728
Cognitive impairment	134 (51.0%)	127 (50.4%)	7 (63.6%)	0.540
Active neoplasia	23 (8.8%)	22 (8.8%)	1 (9.1%)	> 0.999
History of coronary disease	36 (13.6%)	35 (13.8%)	1 (9.1%)	> 0.999
History of stroke	40 (15.2%)	35 (13.8%)	5 (45.5%)	0.014
Atrial fibrillation	61 (23.1%)	58 (22.9%)	3 (27.3%)	0.719
Hypertension	187 (70.8%)	179 (70.8%)	8 (72.7%)	> 0.999
Dyslipidemia	91 (34.5%)	84 (33.2%)	7 (63.6%)	0.051
PE or DVT	6 (2.26%)	6 (2.36%)	0	> 0.999
BMI, kg × m^−2^	24.8 ± 6.4	24.9 ± 6.4	20.7 ± 3.5	0.002
Asymptomatic	22 (8.5%)	19 (7.7%)	3 (27.3%)	0.056
Cough	160 (61.5%)	155 (62.2%)	5 (45.5%)	0.344
Dyspnea	99 (38.1%)	95 (38.2%)	4 (36.4%)	> 0.999
Tiredness	126 (48.5%)	125 (50.2%)	1 (9.1%)	0.010
Falls	30 (11.5%)	28 (11.2%)	2 (18.2%)	0.369
Delirium	53 (20.4%)	49 (19.7%)	4 (36.4%)	0.242
FIM (18–126)	71.8 ± 29.4	72.2 ± 29.7	62.1 ± 20.7	0.222
CIRS-G (0–56)	19.1 ± 6.2	19.0 ± 6.3	20.3 ± 2.3	0.170
CFS ≥5	190 (80.9%)	182 (80.5%)	8 (88.9%)	> 0.999
PSI (0–395)	126.1 ± 53.9	125.9 ± 54.7	130.7 ± 32.3	0.650
CURB-65				0.960
1–2	168 (63.6%)	161 (63.6%)	7 (63.6%)	
3	86 (32.6%)	82 (32.4%)	4 (36.4%)	
4–5	10 (3.8%)	10 (4.0%)	0 (0.0%)	
FiO_2_, %	26.4 ± 9.6	26.5 ± 9.6	24.2 ± 7.5	0.394
Lymphocytes, G/l	1.2 ± 1.3	1.2 ± 1.3	1.0 ± 0.5	0.361
Pro-brain natriuretic peptide, pg/ml	5904.0 ± 10,525.5	5129.4 ± 8216.0	25,657.8 ± 32,622.7	0.297
C-reactive protein, mg/l	67.8 ± 72.0	66.4 ± 71.1	98.1 ± 89.1	0.270

*Abbreviations*: *ADL* activities of daily living, *IADL* instrumental activities of daily living, *COPD* chronic obstructive pulmonary disease, *PE* pulmonary embolism, *DVT* deep vein thrombosis, *BMI* body mass index, *FIM* Functional Independence Measure, *CIRS-G* Cumulative Illness Rating Scale-Geriatric, *CFS* Clinical Frailty Score, *PSI* pneumonia severity index, *FiO*_*2*_ Fraction of inspired oxygen

In an univariate logistic regression model of stroke prediction, active smoking and previous stroke remained significant predictors, increasing by more than seven times and by more than five times, respectively, the risk of stroke. Similarly, each additional point in BMI was associated with a reduced risk of stroke by approximately 14% (Table 2).

**Table 2:** Univariate logistic regression models for stroke predictors

	OR	95% CI	*p* value
Active smoking	7.44	1.75–31.64	0.007
Previous stroke	5.19	1.50–17.92	0.009
Body mass index	0.86	0.74–0.98	0.03

### Stroke characteristics and prognosis

Of the 11 patients with acute stroke, 81.8% were ischemic (9/11) and 18.2% hemorrhagic (2/11). The stroke events occurred after an average of 15.2 days from COVID-19 diagnosis, and the majority of patients presented with stroke during the acute care stay (8/11; 72.7%). Three patients had a simultaneous diagnosis of COVID-19 and acute stroke, whereas another three patients suffered a stroke on a geriatric rehabilitation ward (Supplementary Table S2). An altered state of consciousness and/or delirium were the most frequent clinical manifestations of stroke, reported in 81.8% of cases (9/11). In five patients (45.5%), a focal neurological deficit was present at the time of imaging by CT or MRI. Thromboembolic risk assessed by the CHA2DS2-VASc score showed a median score of 5 (range, 3–7), and a HAS-BLED score of 3 (range, 1–5).

Large vessel occlusion was reported in 22.2% of ischemic stroke cases (2/9). Furthermore, strokes were mainly limited to one side (5/9 right, 3/9 left) and the middle cerebral artery territory was affected in more than half of all cases (5/9; 55.5%), followed by the posterior cerebral artery (3/9), and vertebrobasilar territories (2/9). Ischemic lesions in multiple territories were found in two cases (Supplementary Table S1).

A cardioembolic cause was identified in 3 cases (44.5%), followed by suspected arterio-arterial embolism in 2 cases, and small-vessel occlusion in 1 case. Furthermore, one patient presented a border zone infarct as a consequence of systemic hypoperfusion. Ischemic stroke was classified as cryptogenic in two cases, with no etiology determined in acute workup.

By the end of the hospital stay, three patients (27.3%) died between 3 and 6 days after the stroke occurrence. In-hospital mortality rates were similar between patients with and without acute stroke, as well as institutionalization rates at hospital discharge (21.1% vs 36.4%; *p* = 0.258). All survivors presented moderate to severe disability at discharge (Supplementary Fig. S1).

### Additional features in neuroimaging

There was a high burden of cerebral small vessel disease in patients with stroke, with more than half of patients presenting concomitant lacunes (54.5%). Significant white matter lesions were described in 81.8% of cases and 45.5% of patients had at least one cerebral microbleed, in a classical deep or lobar topography. There was no evidence of features such as meningitis, encephalitis, or vasculitis.

## Discussion

In this study, we report a higher incidence of stroke in a population of old and very old patients with COVID-19, compared to previous descriptions in younger cohorts. However, stroke did not increase the risk of dying, and old and very old patients that survived COVID-19 and an acute stroke had similar outcomes than those without this complication. Importantly, active smoking, previous stroke history, together with a low BMI, were significant predictors of cerebrovascular complications in this age group. Stroke was frequently manifested by delirium and/or altered state of consciousness, and the middle cerebral artery territory was the most frequently affected brain region.

The higher incidence of stroke in this study compared to previous reports is probably the result of the higher age and the consequent higher prevalence of cerebrovascular risk factors. Historic population-based studies demonstrated an early case fatality related to all types of stroke varying from 20 to 30% worldwide [22]. The high rates of overall in-hospital mortality in frail older patients due to COVID-19 possibly overpasses the effect of stroke morbidity alone, explaining the differences regarding a strong association with mortality in younger patients [23]. Long-term follow-up of our patients will clarify whether mortality, institutionalization and rehospitalization rates after hospital discharge will remain similar between groups.

An interesting result of this study was the high prevalence of underweight patients in the stroke group. Although malnutrition is associated with increased length of hospital stay, rehospitalization and mortality after stroke [24], its role as a predictor of stroke occurrence is less well understood, with conflicting evidence [25–27]. Underweight is usually a proxy of malnutrition and sarcopenia, which are possibly related to a higher cardiovascular disease risk [28, 29]. Malnutrition on the other can also be a consequence of a high cardiovascular disease burden, especially in frail older patients [30], in whom a complete nutritional assessment since hospital admission should be part of the clinical workup.

Focal neurological signs were less frequent than delirium as stroke manifestations in this study. The latter may be present in up to 48% of patients with acute stroke [31] and is usually associated with older age, multiple comorbidities and the extent of ischemia [32–34]. Similarly, a decreased level of consciousness and delayed awaking after stopping sedation were associated with a stroke diagnosis in ICU patients, in the absence of focal deficits [35].

A recent grouped analysis of the first 160 stroke cases reported in the course of COVID-19 showed that 76.6% of patients presented stroke in the middle cerebral artery territory. In the subgroup of patients older than 70 years (the oldest patient having had 74.3 years of age), it remained the most frequent territory affected, but dropped to 69% with an increased proportion of vertebrobasilar territory ischemia. We found a similar pattern in our study of still older patients. Importantly, in very old patients, stroke was mostly related to a thromboembolic event and associated with previous risk factors. We found no evidence for a direct neurotropic effect of the SARS-CoV-2 virus, as advocated by other reports [36].

This study has several limitations. Firstly, the cross-sectional analysis of cerebrovascular risk factors allowed us to describe the phenotypes of patients at risk, but not to assess causality. Secondly, our study population represents a very specific population of older patients ineligible for intensive care, thus caution is warranted when extrapolating the results to other settings, especially as far as mortality rates and associated factors are concerned. Ineligibility to intensive care was based on patients’ wishes and took into account a global geriatric assessment considering severity of the disease, underlying comorbidities, and sometimes also consultation of family. Finally, the small number of stroke cases in this study prevented us from calculating multiple variable models of prediction, and some negative findings may be a mere consequence of a lack of statistical power in the analysis.

## Conclusion

In conclusion, clinicians should be aware that incident stroke in patients with COVID-19 is more frequent in old and very old patients, typically manifesting by delirium and decreased level of consciousness, with the middle cerebral artery territory being the most commonly affected. In this study, previous stroke, active smoking, and low BMI were associated with a higher occurrence of stroke in the old and very old age group, and stroke influenced neither the early mortality rate, nor the prevalence of institutionalization by the time of hospital discharge.

## Supplementary Information

**Additional file 1.**

## Data Availability

The datasets used and/or analysed during the current study are available from the corresponding author on reasonable request.
