# Supplementary material for: Incidence, characteristics and clinical relevance of acute stroke in old patients hospitalized with COVID-19
Source: BMC Geriatr. 2021 Jan 14;21:52. doi: 10.1186/s12877-021-02006-2 (PMC7807227; doi:10.1186/s12877-021-02006-2)
Supplement: Supplementary file 1 — Additional file 1. [file 12877_2021_2006_MOESM1_ESM.zip › Supplemental Material_21.12.pdf]

## Supplemental Material

# Incidence, characteristics and clinical relevance of acute stroke in old patients hospitalized with COVID-19

Aline Mendes, MD[1], François R. Herrmann MD, MPH[1], Laurence Genton MD, PhD[2], Christine Serratrice MD[3], Emmanuel Carrera MD[4], Maria Isabel Vargas MD[5], Gabriel Gold MD[1], Christophe E. Graf MD[6], Dina Zekry MD, PhD[3], Max Scheffler MD[7]

[1] Division of Geriatrics, Department of Rehabilitation and Geriatrics, University Hospitals of Geneva and Faculty of Medicine, Geneva, Switzerland.

[2] Unit of Clinical Nutrition, University Hospitals of Geneva and Faculty of Medicine, Geneva, Switzerland.

[3] Division of Internal Medicine for the Aged, Department of Rehabilitation and Geriatrics, University Hospitals of Geneva and Faculty of Medicine, Geneva, Switzerland.

[4] Division of Neurology, Department of Neurosciences, University Hospitals of Geneva and Faculty of Medicine, Geneva, Switzerland.

[5] Division of Neuroradiology, Diagnostic Department, University Hospitals of Geneva and Faculty of Medicine, Geneva, Switzerland

[6] Division of Internal Medicine and Rehabilitation, Department of Rehabilitation and Geriatrics, University Hospitals of Geneva and Faculty of Medicine, Geneva, Switzerland.

[7]Division of Radiology, Diagnostic Department, University Hospitals of Geneva, Geneva, Switzerland

## Supplemental Material

**Supplementary Table S1.** Characteristics of acute ischemic stroke in COVID-19 patients

| | **Large vessel occlusion** | **Infarct limited to the left side** | **Infarct limited to the right side** | **Bilateral infarcts** | **Middle cerebral artery territory** | **Anterior cerebral artery territory** | **Posterior cerebral artery territory** | **Vertebrobasilar territory** | **Multiple territories** | **Confluent white matter lesions** | **Lacunes** | **Cerebral microbleeds** |
|---|---|---|---|---|---|---|---|---|---|---|---|---|
| Case 1 | X | X | | | | | X | | | | X | |
| Case 2 | | X | | | | | | X | | X | | |
| Case 3 | X | | X | | X | | | | | X | | X |
| Case 4 | | | X | | X | | | | | X | X | X |
| Case 5 | | | | X | X | | | | X | X | X | X |
| Case 6 | | X | | | X | | | | | X | | X |
| Case 7 | | | X | | | | X | | | X | X | |
| Case 8 | | | X | | X | X | X | | X | X | X | X |
| Case 9 | | | X | | | | | X | | X | | |

**Supplementary Table S2.** Detailed description of all stroke cases. AF = atrial fibrillation; ADL = activities of daily living

| Cases | Time from COVID-19 diagnosis to stroke (days) | Clinical manifestation | Stroke description | White matter lesions | Cerebral microbleeds | Lacunes | CHA2DS2-VASc (0-9) | HAS-BLED (0-9) | Antithrombotic treatment | Place of living at admission | Destination after discharge | Modified Rankin Scale |
|---|---|---|---|---|---|---|---|---|---|---|---|---|
| 1 | 3 | Right hemiplegia and decreased level of consciousness | Ischemic injury to the territory of the left posterior cerebral artery with occlusion of the latter | Punctate foci | None | Thalamic and left striatal lacunes. | 5 | 2 | None | Nursing home | Deceased 4 days after stroke | 6 |
| 2 | 1 | Left hemiparesis and decreased level of consciousness | Acute ischemia with right lateral pontine hypodensity measuring 13 mm | Large confluent areas | None | Lacune of the right internal capsule | 6 | 4 | None | Nursing home | Deceased 6 days after stroke | 6 |
| 3 | 45 | Left hemiplegia | Acute ischemic lesions of the right superficial and deep middle cerebral artery territories, without hemorrhagic transformation | Large confluent areas | Right hippocampal, left semioval center, and left temporo-occipital | None | 5 | 3 | Aspirin | Home | Home | 3 |

| | | | | | | | | | | | | |
|---|---|---|---|---|---|---|---|---|---|---|---|---|
| 4 | 25 | Delirium | Acute ischemic injury of the right corona radiata | Large confluent areas | Right cerebellar and one left frontal microbleed | Lacunes of the left and right semioval center, of the corona radiata and the left thalamus | 5 | 5 | Aspirin | Home with help in ADL | Nursing home | 5 |
| 5 | 3 | Decreased level of consciousness | Acute ischemic lesions of the left globus pallidus and acute/subacute lesions in the right semioval center with a pre-central cortical component on the right side; no hemorrhagic transformation | Beginning confluence | One left occipital and one left caudate microbleed | Etat lacunaire of the basal ganglia | 7 | 4 | Acenocoumarol (for AF) | Home with help in ADL | Nursing home | 4 |
| 6 | 9 | Delirium | 13 x 3.5 mm parietal lesion, junctional territory lesions of the middle and posterior cerebral arteries | Large confluent areas | One temporal microbleed on the right | None | 4 | 2 | None | Nursing Home | Nursing home | 4 |
| 7 | Concomitant diagnosis | Hemiparesis of the left lower limb and delirium | Ischemia of the right thalamus and right occipital cortico-subcortical region | Beginning confluence | None | Right thalamic lacune | 4 | 1 | None | Home with formal help in ADL | Nursing home | 3 |

| | | | | | | | | | | | | |
|---|---|---|---|---|---|---|---|---|---|---|---|---|
| 8 | Concomitant diagnosis | Left brachio-crural sensory-motor hemisyndrome, with ataxia and heminegligence | Punctiform ischemic lesions of the right cerebral hemisphere concerning the semioval center, the superior parietal gyrus, and the middle frontal gyrus, in addition to multiple lesions of border zones on both sides, with a right predominance | Beginning confluence | One left cerebellar microbleed | Lacunar state with multiple ischemic sequelae of white matter, right basal ganglia, and bilateral thalami | 6 | 4 | Apixaban (for AF) | Home | Home | 4 |
| 9 | Concomitant diagnosis | Delirium with visual hallucinations | Acute ischemic stroke without hemorrhagic transformation of the right cerebellum, measuring 8 x 5 mm | Large confluent areas | None | None | 3 | 2 | None | Home | Home | 3 |
| 10 | 70 | Decreased level of consciousness | Acute left putaminal 9 mm intraparenchymal bleeding | Beginning confluence | None | None | 7 | 4 | Rivaroxaban (for AF) | Home with formal help in ADL | Nursing home | 3 |
| 11 | 11 | Decreased level of consciousness | Right frontal subcortical intraparenchymal hemorrhage | Punctate foci | None | Lacunes of caudate nucleus on the right and head of caudate nucleus on the left | 5 | 4 | Clopidogrel | Home with formal help in ADL | Deceased 3 days after stroke | 6 |

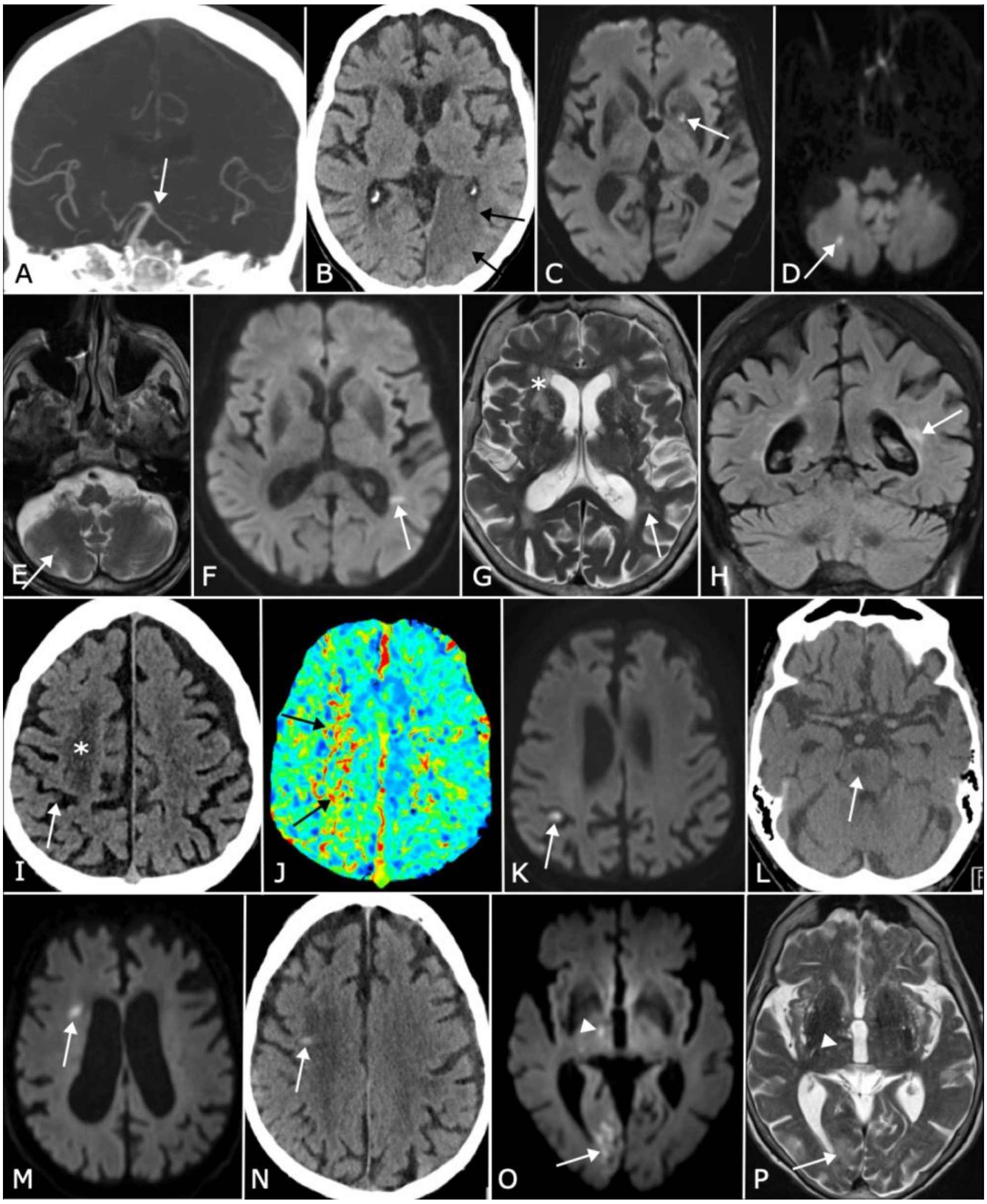


**Supplementary Figure S1.** Supplementary images of vascular neurologic complications in old and very old patients hospitalized with coronavirus disease 2019 infection. *A*, *B*, Computed tomography (CT) images of an 86-year-old woman. *A*, Coronal reconstruction of CT angiography shows occlusion of the left posterior cerebral artery at its origin (arrow). *B*, Noncontrast CT (NCCT) shows hypoattenuating parenchyma, loss of gray-white matter differentiation, and swelling of the left occipital lobe (arrows), consistent with acute ischemia. *C*, Diffusion-weighted imaging (DWI) magnetic resonance imaging (MRI) image of an 89-year-old woman shows small hyperintensity in the left capsulo-lenticular region (arrow), consistent with acute ischemia. *D*, DWI MRI image of 93-year old man shows a small ischemic lesion in right cerebellum (arrow). *E*, T2-weighted image of the same patient shows lesion equally hyperintense, constituted (arrow). *F*, DWI MRI image of an 88-year-old woman shows small ischemic lesion adjacent to left lateral ventricle (arrow). This acute constituted ischemic lesion has hyperintense correlates on T2-weighted (*G*) and coronal fluid-attenuated inversion recovery (*H*) images (arrows). Note areas of significant hyperintense white matter changes in periventricular regions (*G*, asterisk), consistent with microvascular changes. *I*, NCCT image of a 91-year-old man shows small cortical hypodensity in the right parietal lobe (arrow), consistent with an acute ischemic lesion. Subcortical white matter hypoattenuation (*I*, asterisk) is suggestive of

microvascular changes. *J*, Mean transit time color map of perfusion CT image of the same patient shows more extensive area of hypoperfusion in right centrum semiovale (arrows). *K*, Subsequently performed DWI MRI shows cortical ischemic lesion as a focal hypersignal (arrow). *L*, NCCT image of 81-year-old man shows hypodensity in right brainstem (arrow), related to acute ischemia. *M*, DWI MRI image of a 78-year-old man shows a small ischemic lesion in right corona radiata (arrow). *N*, NCCT image of a 91-year-old man shows small hyperdense hemorrhagic focus (arrow) in right centrum semiovale. *O*, DWI MRI image of an 88-year-old woman shows an acute ischemic lesion in the right occipital lobe (arrow) and right thalamus (arrowhead). The lesions are visible as hypersignals on the corresponding T2-weighted image (*P*, arrow and arrowhead).
