# Supplementary figures and images for: Incidence, characteristics and clinical relevance of acute stroke in old patients hospitalized with COVID-19
Source: BMC Geriatr. 2021 Jan 14;21:52. doi: 10.1186/s12877-021-02006-2 (PMC7807227; doi:10.1186/s12877-021-02006-2)

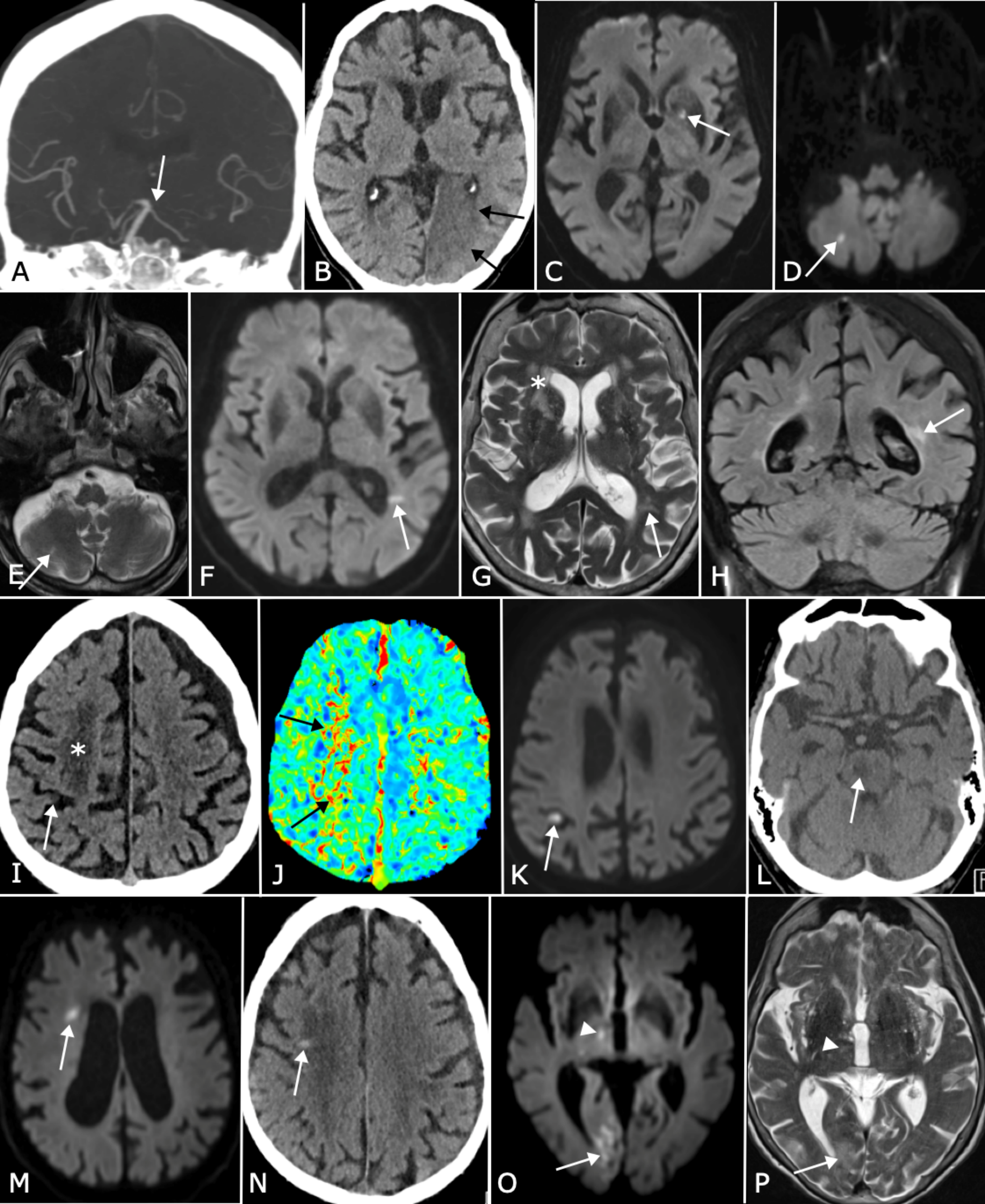

Supplement: Supplementary file 1 — Additional file 1. [file 12877_2021_2006_MOESM1_ESM.zip › Supplementary figure1.pdf]
